# STE20/SPS1-Related Proline/Alanine-Rich Kinase Is Involved in Plasticity of GABA Signaling Function in a Mouse Model of Acquired Epilepsy

**DOI:** 10.1371/journal.pone.0074614

**Published:** 2013-09-13

**Authors:** Libai Yang, Xiaodong Cai, Jueqian Zhou, Shuda Chen, Yishu Chen, Ziyi Chen, Qian Wang, Ziyan Fang, Liemin Zhou

**Affiliations:** 1 Department of Neurology, the 1st Affiliated Hospital, Sun Yat-sen University, Guangzhou, Guangdong, China; 2 Department of Neurology, Shanxi Academy of Medical Sciences & Shanxi Dayi Hospital, Taiyuan, Shanxi, China; 3 Department of Neurology, the 6th Affiliated Hospital, Sun Yat-sen University, Guangzhou, Guangdong, China; Zhejiang University School of Medicine, China

## Abstract

The intracellular concentration of chloride ([Cl^-^]_i_) determines the strength and polarity of GABA neurotransmission. STE20/SPS1-related proline/alanine-rich kinase (SPAK) is known as an indirect regulator of [Cl^-^]_i_ for its activation of Na-K-2 Cl^-^co-transporters (NKCC) and inhibition of K-Cl^-^co-transporters (KCC) in many organs. NKCC1 or KCC2 expression changes have been demonstrated previously in the hippocampal neurons of mice with pilocarpine-induced status epilepticus (PISE). However, it remains unclear whether SPAK modulates [Cl^-^]_i_ via NKCC1 or KCC2 in the brain. Also, there are no data clearly characterizing SPAK expression in cortical or hippocampal neurons or confirming an association between SPAK and epilepsy. In the present study, we examined SPAK expression and co-expression with NKCC1 and KCC2 in the hippocampal neurons of mice with PISE, and we investigated alterations in SPAK expression in the hippocampus of such mice. Significant increases in SPAK mRNA and protein levels were detected during various stages of PISE in the PISE mice in comparison to levels in age-matched sham (control) and blank treatment (control) mice. SPAK and NKCC1 expression increased *in vitro*, while KCC2 was down-regulated in hippocampal neurons following hypoxic conditioning. However, SPAK overexpression did not influence the expression levels of NKCC1 or KCC2. Using co-immunoprecipitation, we determined that the intensity of interaction between SPAK and NKCC1 and between SPAK and KCC2 increased markedly after oxygen-deprivation, whereas SPAK overexpression strengthened the relationships. The [Cl^-^]_i_ of hippocampal neurons changed in a corresponding manner under the different conditions. Our data suggests that SPAK is involved in the plasticity of GABA signaling function in acquired epilepsy via adjustment of [Cl^-^]_i_ in hippocampal neurons.

## Introduction

Mesial temporal lobe epilepsy (MTLE) is recognized as one of the most medically intractable forms of epilepsy. However its pathogenesis remains unclear. Diverse brain insults, including status epilepticus (SE) and stroke, can induce epileptogenesis, a process by which normal brain tissue becomes altered and capable of generating spontaneous recurrent seizures (SRSs) [1]. One of the most important factors contributing to epileptogenesis is the persistent increase in intracellular chloride concentration ([Cl^-^]_i_), which induces a long-lasting shift in the action of g-aminobutyric acid (GABA) in the direction of depolarizing, ultimately leading to seizure generation [2]. The [Cl^-^]_i_ determines the strength and polarity of GABA-mediated neurotransmission [3]. In the mature brain, GABA exerts a hyperpolarizing inhibitory effect as a result of low [Cl^-^]_i_ levels. However, in the immature brain or under pathological conditions, GABA exerts a depolarizing excitatory effect due to excessive intracellular accumulation of Cl^-^. Under such conditions, drugs such as benzodiazepines and phenobarbital exhibit reduced efficacy because GABA receptors are also binding sites for these drugs [4]. Recently, it was discovered that chloride homeostasis can be regulated by several factors, including endogenous modulators [5]. Therefore, chloride homeostasis has become an attractive target for the treatment of central nervous system (CNS) disorders.

The [Cl^-^]_i_ is determined, in part, by the activities of the SLC12 cation-chloride co-transporters (CCCs) which are direct regulators of [Cl^-^]_i_ [6]. In the brain, the transporters include mainly the Na-K-2Cl co-transporter NKCC1, which mediates chloride influx, and the K-Cl^-^co-transporter KCC2, which extrudes chloride from the cell. Thus, abnormal expression or dysfunction of NKCC1 and/or KCC2 may result in altered chloride homeostasis [3]. It has been reported that abnormal expression of CCCs or related functional changes in GABAergic neurons occur in MTLE [7,8] and in pilocarpine-induced status epilepticus (PISE) [9,10,11,12], which suggesting that CCCs are involved in the development of intractable epilepsy. Ischemia can also induce expression changes in NKCC1 and KCC2 expression in hippocampal neurons, which in turn can alter the neuronal response to GABA from hyperpolarization (inhibition) to depolarization (excitation).

The role of the WNKs-SPAK/OSR1-CCCs signaling pathway in regulating CCCs is well documented [13,14,15,16,17,18]. In kidney, intestines and other organs, SPAK (STE20/SPS1-related proline/alanine-rich kinase) is known as an indirect regulator of [Cl^-^]_i_ for its activation of NKCC(Na-K-2Cl^-^co-transporters) and inhibition of KCC (K-Cl^-^co-transporters). However, it remains unclear whether SPAK modulates [Cl^-^]_i_ through NKCC1 or KCC2 in the brain. Also, there are no data clearly establishing SPAK expression in cortical or hippocampal neurons, or establishing an association between SPAK and epilepsy. Because there is no effective intervention targeting NKCC1/KCC2，it is important to explore a new therapeutic target, such as SPAK, for MTLE.

In the study described herein, after confirming SPAK expression in mouse hippocampal neurons and its co-expression with CCCs NKCC1 and KCC2, we studyed the long-term expression profile of SPAK in the hippocampus of PISE -affected mice. In further experiments, we induced SPAK overexpression and oxygen-deprivation in cultured hippocampal neurons of neonatal mice to explore the intrinsic relations between SPAK and NKCC1and between SPAK and KCC2 that are involved in adjustment of [Cl^-^]_i_. Understanding these relations may provide new clues to understanding the pathogenesis of acquired epilepsy. We speculate that SPAK is involved in the plasticity of the GABA signaling function in epileptogenesis through adjustment of [Cl^-^]_i_ in mouse hippocampal neurons. Therefore, SPAK may be a novel target for the treatment of epilepsy.

## Materials and Methods

### Ethics Statement

The study was carried out in strict accordance with the Guide for the Care and Use of Laboratory Animals of the National Institutes of Health. The protocol was approved by the Animal Care Committee of Sun Yat-sen University. All surgeries were performed under chloral hydrate following previous study in PISE model [11,19,20], and all efforts were made to minimize suffering at each stage of the experiments.

### Animals, status epilepticus induction

One hundred and eight healthy male Balb/c mice (18~22g, 6~8 weeks) were used in the study. The animals were randomly divided into three groups: a PISE group, a sham-control group and a blank-control group.

PISE was induced by intraperitoneal (ip) injection with lithium-pilocarpine as described previously [9,21]. In brief, mice in the PISE group were injected with pilocarpine (280~340 mg/kg ip, Sigma, China) 24 h after ip administration of lithium chloride (127 mg/kg, Sigma, China). Pilocarpine doses greater than 340 mg/kg were avoided because of an increased risk of death. To counteract the peripheral cholinergic effects of pilocarpine and reduce the risk of death due to respiratory insufficiency, methyl-scopolamine bromide (1 mg/kg ip, Sigma, China) was administered 30 min before the administration of pilocarpine. SE was defined as continuous limbic seizure activity and was interrupted after 1 h by administration of diazepam (0.1 mg/kg ip, Sigma). All experimental animals received two injections of saline (0.5 mL ip), one immediately after SE interruption and one on the day after SE induction to prevent dehydration. Sham-control mice received saline instead of pilocarpine. Nothing was administered to the blank-control mice.

The evoked behavioral seizures were classified according to Racine’s standard criteria [22], i.e., stage 1, immobility, staring; stage 2, rigid posture; stage 3, repetitive movements, head bobbing; stage 4, rearing, myoclonic twitching; stage 5, severe tonic-clonic seizures. Only those animals that reached stages 4 of 5 were taken into consideration. Three main phases were observed following PISE: the acute stage, the subacute stage, and the chronic stage with spontaneous recurrent seizures, represented as day 1, day 14 and day 45 respectively, after PISE. Six mice were allocated to each time point in all three groups.

### Tissue Preparation

Half of the mice in each subgroup were used for Western blotting and quantitative RT-PCR, and the others were used for immunohistochemistry. Mice were decapitated following anesthesia with 10% chloral hydrate (3.5 mL/kg, intraperitoneally) and perfused with 0.9% saline at 4°C alone, or followed by fixation with 4% cold paraformaldehyde in phosphate buffer (0.1 mol/L, pH 7.4), depending on the intended use of the tissue. Both sides of the hippocampus and the whole brain were isolated on an ice plate. Hippocampi were snap frozen in liquid nitrogen and stored at −80 °C for RT-PCR and Western blotting. Whole brains were sectioned for immunohistochemistry.

### Real-time quantitative PCR

The hippocampus was isolated from the mouse brain, weighed (20mg) and homogenized for total RNA collection with Trizol (Invitrogen Technology, Carlsbad, CA, USA). SPAK mRNA expression was detected by real-time quantitative PCR. An initial strand of cDNA was synthesized from 1 mg of total cellular RNA with random 6mers with the ExScript™\RT reagent kit (Takara Biotechnology, Dalian, China). RT-PCR cycles were carried out for amplification of SPAK and GAPDH with a DNA Engine Opticon Continuous Fluorescence Detection System (DFC-3200, MJ Research Company, USA). Two microliters of cDNA and 1 µL primer were used in a final 20-µL reaction solution with SYBR Green Realtime PCR Master Mix (QRT-201, Toyobo, Osaka, Japan). The annealing temperature was 58°C in the PCR reaction, as the specific product of SPAK was amplified at 58°C, whereas GAPDH could be amplified at a wider temperature range. Primers were designed and synthesized by Takara. The sequences are as follows:

GAPDH (GenBank No: NM_001001303):
Forward primer: 5’- TGTGTCCGTCGTGGATCTGA -3’
Reverse primer: 5’- TTGCTGTTGAAGTCGCAGGAG -3’
SPAK (GenBank No: NM_016866):
Forward primer: 5’- CAGATCTCAAACCTGCACTACACGA -3’
Reverse primer: 5’- ACAGAACGGCAGCAAGGTTACA-3’

The PCR products for SPAK and GAPDH were confirmed by sequencing. The PCR products were quantified in relative terms by means of the double-standard curve method and are expressed as arbitrary units.

### Western blot analysis

Total protein was extracted from the hippocampus and from cultured neurons. Protein concentrations were determined with the Micro BCA Protein Assay Kit (23235, Pierce, USA). After SDS-PAGE gel electrophoresis on a 5% stacking gel and 8% (for SPAK) or 6% (for NKCC1, KCC2) separating gel, proteins were transferred to a polyvinylidene fluoride (PVDF) membrane (Millipore Corporation, MA, USA). The membrane was blocked in 5% fresh non-fat milk (Amresco, Solon, OH, USA) for 1 h at room temperature with gentle shaking and incubated with primary antibodies rabbit anti-SPAK(1:200, AP7968c, Abgent, China.), goat anti-NKCC1 (1:200, sc-21547 Santa Cruz Biotechnology, USA) and goat anti- KCC2(1:200, sc-19420, Santa Cruz Biotechnology) and mouse anti-β-actin (1:10000, Mab1445, Sigma-Aldrich, USA) overnight at 4°C followed by TBST washes and incubation for 60 min at room temperature in TBST containing appropriate HRP-conjugated secondary antibody (00001-1, Proteintech Group, China, 1:1000 for SPAK: 00001-2, Proteintech Group, 1:2000 for β-actin: sc-2020, Santa Cruz Biotechnology, 1:2000 for NKCC1and KCC2). Immunodetection of proteins by chemiluminescence (7003, Cell Signaling Technology, USA) was followed by exposure to X-ray film, with sufficient time allowed for each antibody. PVDF membrane was also used for detecting proteins for SPAK or NKCC1, KCC2, and β-actin. β-actin was used for equal loading control. The size of proteins was determined by running a BenchMark™Prestained Protein Ladder (Invitrogen Technology).

### Immunochemistry

Ten-micrometer-thick sections were cut on a cryostat (2800N, Leica, Germany). Immunohistochemistry was performed according to the Envision immunohistochemical technique, as described previously [23]. Briefly, sections were pretreated for 10 min with hot (85°C) 0.01 mol/L citrate buffer (pH 6.0), rinsed in phosphate-buffered saline (PBS) three times for 5 minutes each, then treated with 3% hydrogen peroxide for 10 minutes before being rinsed again three times in PBS. The sections were incubated with the primary antibody rabbit anti-SPAK (1:200, AP7968c, Abgent, China) overnight at 4°C. After three additional washes in PBS, the sections were incubated with ready-to-use peroxidase-marked rabbit/mouse secondary antibody (K5007, Dako, Glostrup, Denmark) for 1 hr at room temperature. The signal was visualized with 3,3-diaminobenzidinetetrahydrochloride (DAB, Dako). Negative-control sections were incubated with PBS instead of the primary antibody, and they showed no positive signal.

A similar protocol was used in immunofluorescence for determining SPAK co-expression with neuronal nuclei (NeuN), a specific marker of neurons, NKCC1, and KCC2. Sections were incubated in 0.01M PBS containing 0.3% Triton-X 100 for 20 min before addition of primary antibody, and sections were incubated with anti-SPAK (1:100, AP7968c, Abgent) together with the primary antibodies mouse anti-NeuN (1:1000, MAB377, Millipore, Bedford, MA, USA), goat anti-NKCC1 (1:100, sc-21547 Santa Cruz Biotechnology) or goat anti-KCC2(1:100, sc-19420, Santa Cruz Biotechnology). The secondary antibodies used were FITC-conjugate goat anti-rabbit IgG (1:200, AP132F, Chemicon International, USA), Cy3-conjugated goat anti-mouse IgG (1:400, AP124C, Chemicon International, USA), or Cy3-conjugated donkey anti-goat IgG (1:100, SA0009-3, Proteintech Group). Fluorescent signals were detected with a BX51 microscope (Olympus, Tokyo, Japan) at excitation/emission wavelengths of 495/519 nm (FITC, green) and 550/570 nm (Cy3, red).

### Primary culture of hippocampal neurons

Mouse hippocampal neurons were isolated and cultured according to a published protocol [24], with slight modifications. Briefly, hippocampi were dissected out from the brains of neonatal mice younger than 24 hours in ice-cold HBSS (C14175, Gibco, USA) and the meninges around them were removed. After being cut into pieces, the tissues were incubated in 0.08% trypsin (25200, Gibco) in a water bath at 37°C for 15 min. The trypsin was removed, and the hippocampi were dissociated in HBSS by repeated pipetting up and down in a Pasteur pipette. The medium was replaced with Neurobasal medium (10888,Gibco) containing B27 supplement (0113, Gibco) and L-Glutamine (25030081, Gibco). Cells were seeded on polylysine-laminin-coated (P1274-25MG, Poly-L-Lysine hydrobromide Sigma-Aldrich, and 23017-015, Natural Mouse Laminin, Invitrogen Technology) coverslips (2000–5000 cells ⁄cm^2^) or 35 mm dishes, and cultured in a humidified incubator (Thermo Electron Corporation, USA) containing 5% CO_2_ at 37 °C. The medium was half-changed every 3 days after plating until ready to use.

### SPAK overexpression

Overexpression of SPAK in neurons was established by lentiviral vector pGC-FU-Stk39-GFP (constructed by Shanghai GeneChem Co., Ltd., China) transformation. Twelve hours after seeding, neurons were infected with pGC-FU-Stk39-GFP by being cultured in medium containing lentivirus. Ten hours later, the medium was replaced with normal medium. The multiplicity of infection (MOI) was 5. The optimum infection time and MOI values noted above were determined according to the results of MTT assay and infection efficiency under various infection conditions. Negative lentiviral vector infection and non-infection were used for negative controls.

### Oxygen deprivation

Oxygen deprivation was performed on day 10 of hippocampal neuron culture. Hypoxic conditioning was achieved by culturing the cells in an anaerobic chamber that was flushed with a gas mixture of 5% CO_2_ and 95% N_2_ (v/v) at 37°C for 30 min [25]. The cells were cultured in the incubator described above for another 24h before any measurements were performed. All of the procedures were adopted to simulate the analogic pathophysiologic process of SE *in vivo*.

### Immunoprecipitation and immunoblot analysis

Cells were lysed and harvested in 1 mL of lysis buffer containing 100 mM NaCl, 50 mM NaF, 50 mM Tris-HCl, pH 7.4, 1% Nonidet P-40, 0.25% sodium deoxycholate, 1 mM EDTA, 1 mM EGTA, and a protease inhibitor mixture. SPAK was immunoprecipitated from 500 µg protein by overnight incubation under constant rotation at 4 °C with the use of 4 µg polyclonal antibody to SPAK (AP7968c, Abgent.1:100). Immune complexes were retrieved with 20 µL Protein L-Agarose beads (sc-2336, Santa Cruz Biotechnology, USA) through incubation together under constant rotation at 4 °C for 4 hrs, washed three times with cold PBS, resuspended in 20 µL 2× loading buffer and heated in boiling water for 5 min before centrifugation. The supernatant was subjected to gel electrophoresis as described previously. Protein bands were transferred to PVDF membrane for immunoblot analysis. NKCC1 and KCC2, the potential co-immunoprecipitated proteins, and SPAK were detected by Western blot analysis with specific antibodies and enhanced chemiluminescence. The interaction intensity between SPAK and NKCC1 or KCC2 was represented by the ratio between the values from densitometric analysis.

### Fluorometric measurement of [Cl^-^]_i_

Cultured hippocampal neurons were incubated for 1 h with 10 mM N-(6-methoxyquinolyl) acetoethyl ester (MQAE), a Cl^-^-sensitive fluorescent dye whose fluorescence intensity inversely correlates with the Cl^-^ concentration, and then rinsed and perfused with modified Krebs’ solution (in mM: NaCl 128, KCl 2.5, CaCl2 2.7, MgSO4 1.0, Glucose 16, N-2-hydroxyethylpiperazine-N’-2-ethanesulfonic acid (HEPES) 20, pH adjusted to 7.4 by NaOH). The fluorescence intensity was recorded by laser scanning confocal microscope (ZEISS LSM 510-META confocal microscope, Germany) with excitation and emission wavelengths of 355–365 nm and 450–550 nm, respectively [26,27,28].

The [Cl^-^]_i_ was estimated by calibrating the MQAE fluorescence intensity to [Cl^-^]_i_ as described elsewhere [26,27,28]. Calibration was performed by adding a mixture of ionophores including10 mM valinomycin(385120100, Acros Organics, Belgium), 5 mM nigericin(M02175, Fluorochem Ltd., USA), and 10 mM tributyltin at various [Cl^-^]_i_ in KNO3 solution. The sample neuronal [Cl^-^]_i_ was obtained by means of the Stern Volmer equation F_0_/F=1+Ksv[Cl^-^]_i_ (F_0_, MQAE fluorescence with ionophore and zero bath chloride; F, corresponding MQAE fluorescence; Ksv, the Stern-Volmer constant). In the process, the fluorescent signals of MQAE and GFP overlap as the cells are transfected with a GFP-containing vector. To exclude bias resulting from the interference of GFP fluorescence, neurons infected with a negative lentiviral vector were used as negative controls.

### Analysis

All *in vitro* experiments were repeated independently three or more. Images were analyzed by means of commercial image analysis software (Image Pro Plus 4.5, Media Cybernetics, Silver Spring, MD, USA). The data are shown as the mean ± SD and were analyzed with the use of SPSS 13.0 software. Between-group differences were subjected to one-way ANOVA (analysis of variance), two-way ANOVA followed by a Bonferroni’s post-hoc analysis of multiple comparisons. The statistical graphs were prepared with the use of Microsoft Office Excel 2003 software. A probability value less than 0.05 was considered statistically significant.

## Results

### Behavioral analysis

In this mouse model of epilepsy, the onset of continuous SE was observed at 20.7 ± 6.5 min after pilocarpine injection. SRSs consisted of periods of freezing, clonic forelimb movements, and rearing. Even falling was observed after an average 13.7 ± 5.3-day latency period and occurred in all pilocarpine-treated animals. The duration of spontaneous seizures was typically 10^~^20 s. PISE was successfully established in about 76% of animals, the other mice died of violent convulsions.

### SPAK expression and co-expression with CCCs in mouse hippocampal neurons

**Figure 1:**
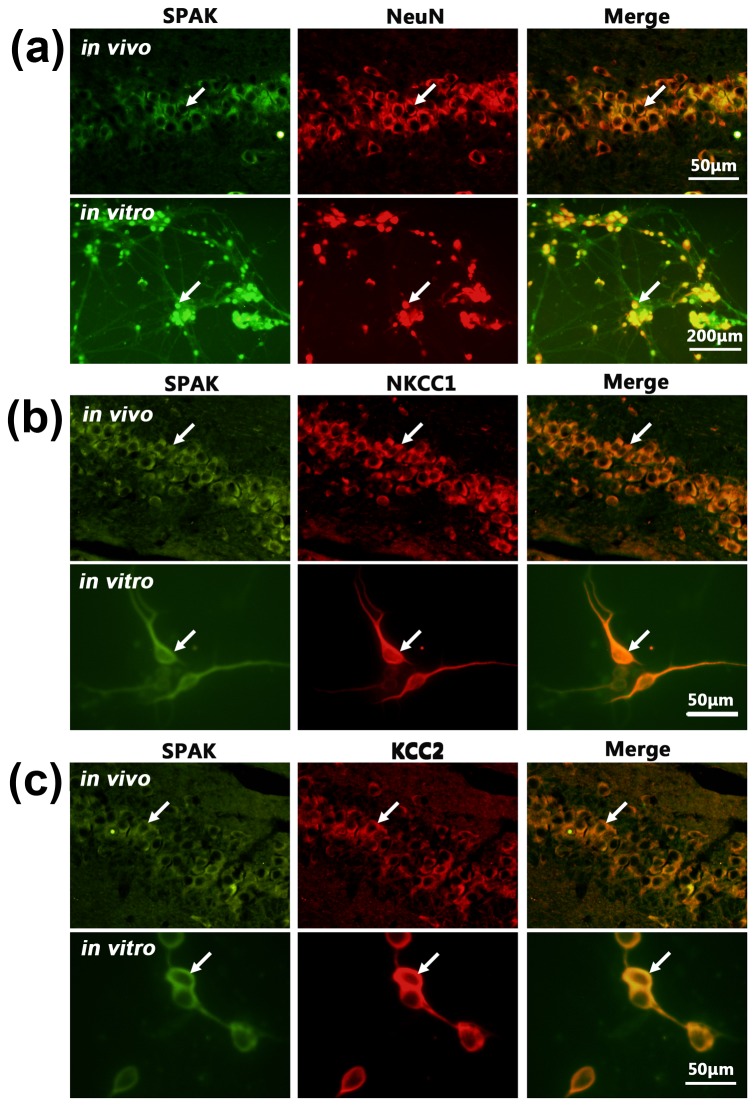
SPAK expression and co-expression with NKCC1 or KCC2 in hippocampal neurons. (a) SPAK co-expression with NeuN. (b) SPAK co-expression with NKCC1. (c) SPAK co-expression with KCC2. *In* *vivo* shows them in mouse hippocampus neurons. *In* *vitro* shows them in cultured hippocampal neurons (arrows indicate positive cells).

In our study, SPAK expression and co-expression with NKCC1 or KCC2 in the mouse hippocampus were prerequisites for further investigation. Immunohistochemistry was performed to establish whether these conditions were met. Immunofluorescence staining showed SPAK co-expressed with NeuN in both the adult mouse hippocampal and neonatal mouse hippocampal neurons *in vitro*, confirming SPAK expression in neuronal populations (Figure 1a). Furthermore, co-expression of SPAK with NKCC1 or KCC2 in adult mouse hippocampal and cultured hippocampus neurons was confirmed (Figure 1b or c).

### SPAK expression increased after PISE

SPAK expression tended to increase in the mouse hippocampus. This increase was followed by retroposition after PISE, corresponding overall to the acute, subacute, and chronic stages represented by day 1, day 14, day 45 after induction of SE.

**Figure 2:**
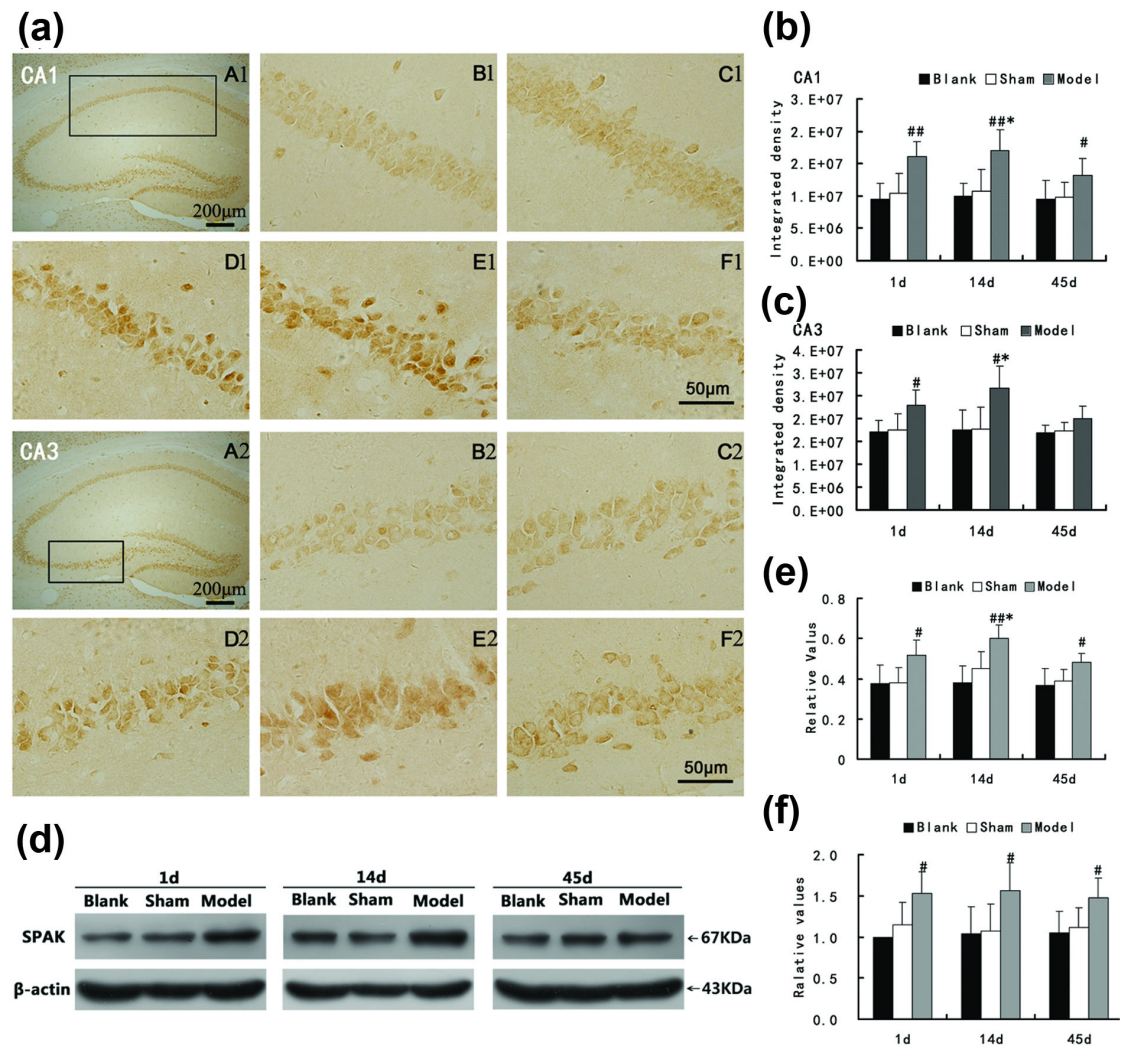
Expression of SPAK in mouse hippocampus at various stages of PISE. (a) (b), (c) Evaluation of SPAK expression in the CA1 and CA3 regions of mice hippocampi after PISE by immunohistochemistry. A: CA1 or CA3 region of hippocampus indicated with a box. B-F: The region selected in “A” from the blank group (B), sham group (C), day after PISE (D), day 14 after PISE (E), and day 45 after PISE (F). (d) (e) Evaluation of SPAK protein expression in mice hippocampi after PISE by Western blotting analysis. (f) Evaluation of SPAK mRNA expression in mice hippocampi after PISE by RT-PCR. Values are mean ± SD, ^#^ *P*< 0.05 versus the blank and sham groups, ^##^ *P*<0.05 versus the blank sham groups, * *P*<0.05 versus day 1 and day 45 in the PISE-affected group. Scale bars in “F” apply to B-F.

In the hippocampal CA1 area, immunohistochemistry showed that the expression of SPAK to be significantly increased on day 1 after SE (Figure 2a A1-F1 and b; *P*<0.01) and then further increased by day14 in comparison to that in the age-matched control groups (*P*<0.01). A downward trend in SPAK expression was observed by day 45, but the expression level was still higher than in control animals at this time point (*P* <0.05). Expression of SPAK on day 14 was higher than on day 45 in the PISE group (*P*<0.05). As in the CA1 area, SPAK expression in the CA3 area was significantly increased on day 1 and day 14 after SE in comparison to that in the age-matched control mice (Figure 2a A2-F2 and c, *P*<0.01). SPAK increase, but the level on day 45 did not differ statistically from that in the control mice (*P*=0.093), although image analysis suggested the level was higher than in the age-matched controls. Expression on day 14 was also higher than on day 45 in the PISE group (*P*<0.05).

Upon Western blot analysis, SPAK was identified as an immunopositive band with a molecular weight of 67 kDa, as expected (Figure 2d). Quantitative analysis suggested significant that alterations in hippocampus SPAK protein expression occurred at various time points following PISE. SPAK immunoreactivity was significantly increased in hippocampal homogenates on day 1, day 14, and day 45 after PISE compared to that in age-matched controls (Figure 2d and e, *P*<0.05). In addition, SPAK expression on day 14 in the PISE group was higher than that observed on day 1 and on day 45, and in comparison to that on day 45, the increase was statistically significant (Figure 2d and e, *P* <0.05).

The expression profile of SPAK mRNA was shown by real-time fluorescence quantitative PCR to be the same as that of protein. Compared to that in age-matched control mice, SPAK mRNA expression in the hippocampus of PISE-affected mice was significantly increased on day 1, day 14, and day 45 after PISE (Figure 2f, *P*<0.05), especially on day 14. However, the differences between the three PISE groups were not statistically significant (Figure 2f, *P*=0.871). The various detection methods did not reveal differences in SPAK expression differences between time points in the blank-control mice hippocampus (Figure 2b, c, e and f, *P*>0.05), which indicated that SPAK expression levels were not significantly influenced by age.

### Alterations in SPAK and CCC expression in primary cultured hippocampal neurons after SPAK overexpression and/or oxygen deprivation

**Figure 3:**
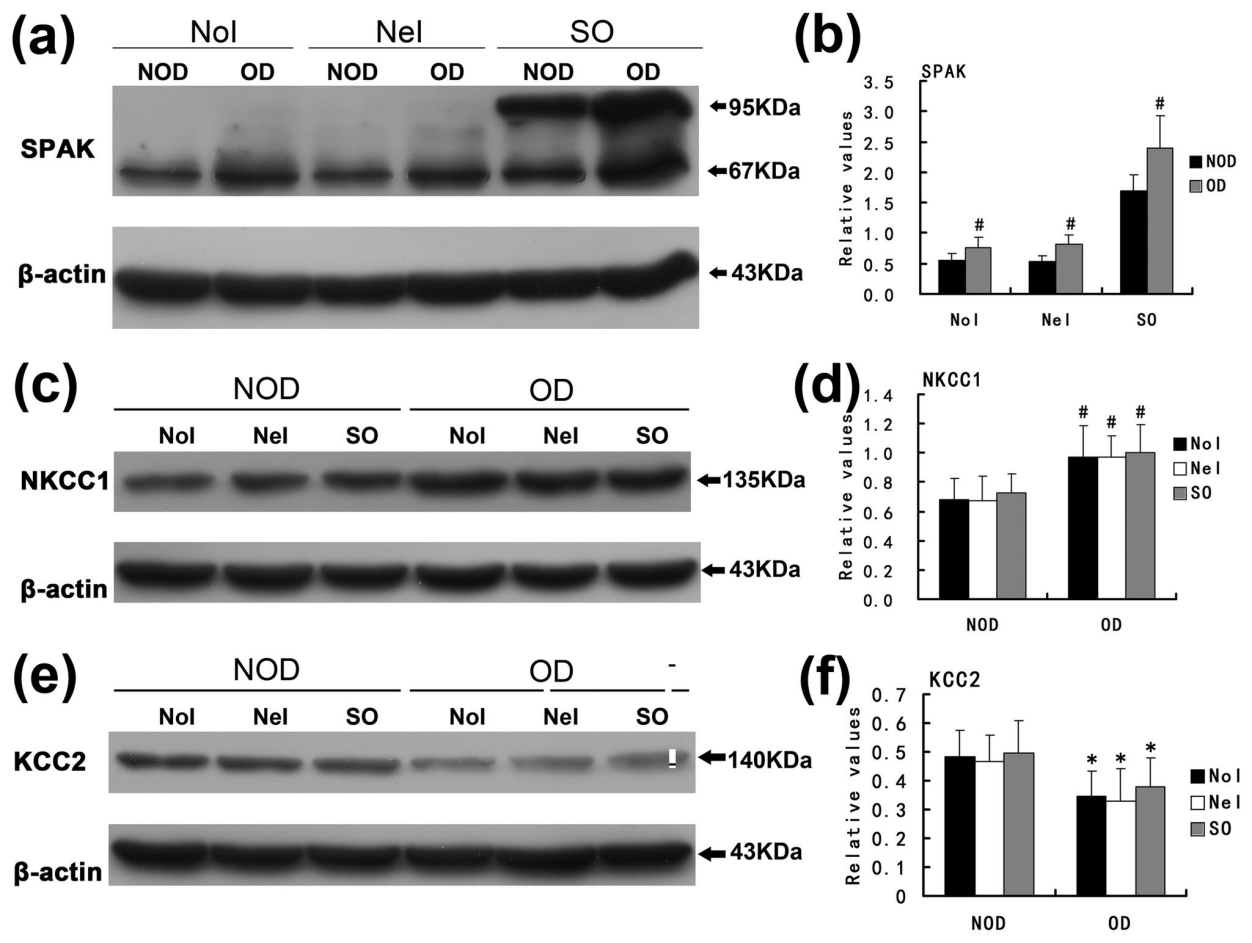
Alterations in expression of SPAK and CCCs in cultured hippocampal neurons after SPAK overexpression and/or oxygen deprivation. (a), (b) SPAK expression level increases after oxygen deprivation in various groups. The bands with molecular weights of 67 kDa and 95 kDa represent endogenous and exogenous SPAK, respectively. (c) (d) NKCC1 expression level increases after oxygen-deprivation in various groups. NKCC1 expression level does not change following SPAK overexpression in any condition. (d) (e) KCC2 expression declines after oxygen-deprivation in various groups. KCC2 expression levels do not change following SPAK overexpression in any condition. NoI: non-infection group. NeI: negative infection group. SO: SPAK overexpression group. OD: oxygen-deprivation. NOD: non-oxygen-deprivation. Values are mean ± SD, ^#^ *P*<0.05 versus the non-oxygen-deprivation group.

Western blotting detected an immunopositive band of 67 kDa in non-infected cultured primary hippocampal neurons and those in the negative lentiviral infection group. However, two bands with molecular weights 67 kDa and 95 kDa were observed in the pGC-FU-Stk39-GFP infection group (Figure 3a). Because the band of 67 kDa is representative of endogenous SPAK, the 95 kDa (molecular weight of GFP is 28kDa) band represents expression of exogenous SPAK. This result further confirmed SPAK expression in hippocampal neurons of neonatal mice and also demonstrated the success of pGC-FU-Stk39-GFP lentiviral infection of primary cultured neurons for achieving SPAK overexpression.

The expression levels of SPAK increased significantly after oxygen-deprivation (versus no oxygen deprivation) in various groups (in the non-infected or negative infection control groups and the pGC-FU-Stk39-GFP infection group) (Figure 3a and b, *P*<0.01). In the pGC-FU-Stk39-GFP group, both endogenous and exogenous SPAK expression increased (Figure 3a and b *P*<0.01). These results can be interpreted as hypoxia-enhanced SPAK expression in hippocampal neurons *in vitro*. The protein expression levels of NKCC1 (molecular weight of 170 kDa) increased significantly (Figure 3c and d), while KCC2 (molecular weight of 140 kDa) decreased significantly after oxygen-deprivation (Figure 3e and f). The differences were statistically significant in both the non-infected and negative lentiviral infection groups and in the pGC-FU-Stk39-GFP group (Figure 3a-f *P* <0.01). With and without oxygen-deprivation, SPAK overexpression failed to influence the expression of NKCC1 (Figure 3c and d, *P*_*NOD*_=0.464, *P*_*OD*_=0.927) or KCC2 in primary cultured hippocampal neurons (Figure 3e and f, *P*_*NOD*_=0.833, *P*_*OD*_=0.585).

### The effect of SPAK overexpression and/or oxygen deprivation on the interaction between SPAK and CCCs in primary hippocampal neurons

**Figure 4:**
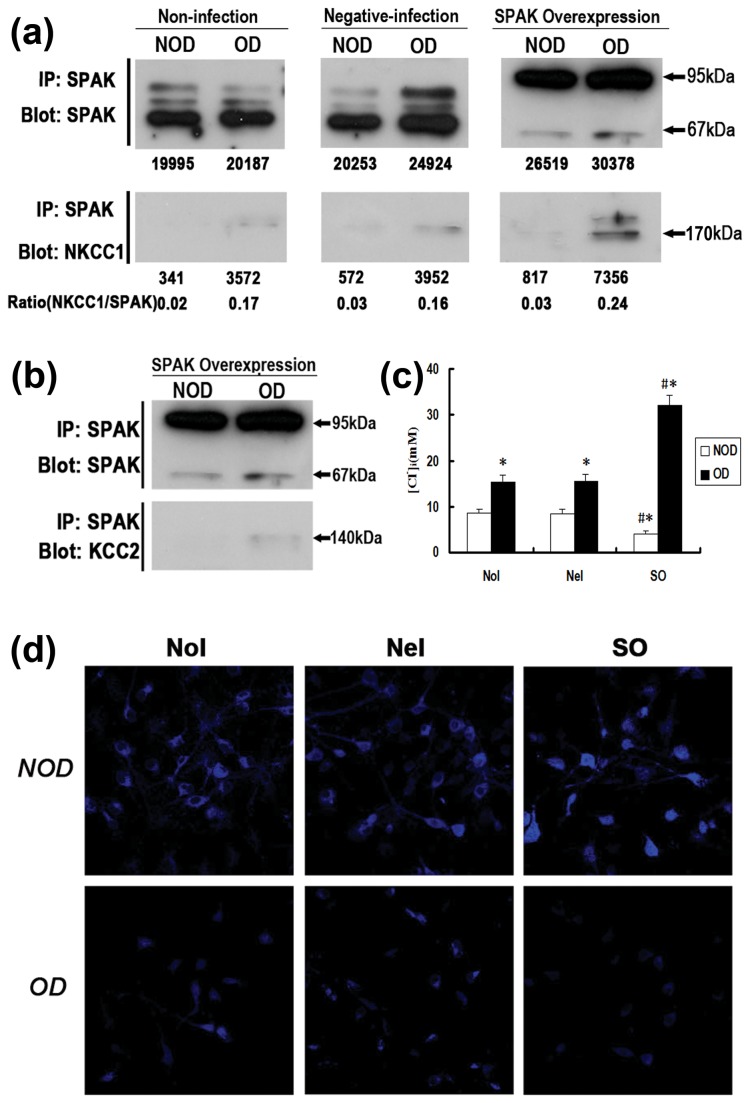
Interaction of SPAK with CCCs and [Cl^-^]_i_ in cultured hippocampal neurons at various stages. (a) (b) The intensity of interaction between SPAK and NKCC1 or KCC2 increased markedly after oxygen deprivation, while SPAK over-expression strengthened the interactions. (c) (d) The [Cl^-^]_i_ of hippocampus neurons depict the corresponding and interesting changes in differing conditions. NoI: non-infection group. NeI: negative infection group. SO: SPAK overexpression group. OD: oxygen-deprivation. NOD: non-oxygen-deprivation. ^#^ *P*<0.05 versus non-infection group and negative infection group, * versus non-oxygen-deprivation group.

Co-IP was used to determine whether SPAK interacts with NKCC1 or KCC2. The proteins pulled down with SPAK are the proteins that are bound to and interacted with SPAK. As illustrated in Figure 4a, oxygen deprivation increased NKCC1 immunoprecipitation with SPAK in primary cultured hippocampal neurons. SPAK over expression failed to enhance the amount of NKCC1 bound to SPAK in the in cultured neurons of the non-oxygen deprived group in comparison to that in the non-infected and negative lentiviral infection groups. However, as the amount of SPAK precipitation increased, more NKCC1 was detected in the pGC-FU-Stk39-GFP-infected group than in the other groups when neurons were oxygen deprived. The NKCC1/SPAK ratio, which represents the intensity of interaction between the 2 proteins, followed a trend expected intuitively. However, KCC2 was detected via co-IP only in nerons with both pGC-FU-Stk39-GFP infected and oxygen deprived (Figure 4b).

### Alterations in [Cl^-^]_i_ after SPAK overexpression and/or oxygen-deprivation

MQAE fluorescence helped detect significant changes in the Cl^-^ concentration in the cultured hippocampal neurons following SPAK overexpression and/or oxygen deprivation (Figure 4c and d and Table 1). The fluorescence intensity increased and [Cl^-^]_i_ decreased in the pGC-FU-Stk39-GFP neurons compared with the control groups under normal conditions (*P*<0.05). These findings suggest [Cl^-^]_i_ decreased in neurons overexpressing SPAK. However, the decreased fluorescence intensity and increased [Cl^-^]_i_ in non-infected and negative lentiviral infection groups after oxygen-deprivation (*P*<0.05) indicated that the lack of oxygen promoted the increase of [Cl^-^]_i_ in hippocampal neurons. Moreover, after hypoxic conditioning, the fluorescence intensity further weakened and [Cl^-^]_i_ further increased in the pGC-FU-Stk39-GFP group compared to that in the control groups（*P*<0.05). Thus, oxygen-deprivation combined with SPAK overexpression resulted in the highest [Cl^-^]_i_ levels in hippocampal neurons.

**Table 1:** Results of MQAE assay.

**Preparation**	**F**	**F_0_**	**F_0_/F**	**[Cl^-^**]**_i_** (mmol/L)
NOD+Nol	14.25±2.68	20.48±3.82	1.44	8.58±0.89
NOD+Nel	14.32±2.56	20.32±2.95	1.42	8.34±1.08
NOD+SO	18.48±2.13	22.38±2.87	1.21	4.11±0.68
OD+Nol	10.86±2.38	19.21±3.68	1.77	15.29±1.56
OD+Nel	11.30±2.32	20.20±4.30	1.78	15.47±1.51
OD+SO	7.21±1.52	18.95±3.34	2.63	32.24±2.06

MQAE: N-(6-methoxyquinolyl) acetoethyl ester; F: corresponding MQAE fluorescence; F_0_: MQAE fluorescence with ionophore and zero bath chloride; Ksv, Stern–Volmer constant; [Cl^-^]_i_: intracellular chloride concentration calculated from the Stern–Volmer equation ([Cl^-^]_i_ =(F_0_/F-1)/ Ksv; K= 0.051 mmol^-1^); NOD: non-oxygen deprivation; OD: oxygen deprivation; NoI: non-infection group; NeI: negative infection group; SO: SPAK overexpression group.

## Discussion

In the present study, we confirmed SPAK expression and co-expression with the CCCs in mouse hippocampal neurons. Subsequently, the up-regulation of SPAK at both the gene and protein level was examined in the hippocampus at various stages following induction of PISE in mice. Further experiments showed that SPAK expression co-altered with NKCC1 and KCC2 in primary cultured hippocampal neurons under hypoxic conditions, whereas SPAK overexpression did not influence expression of NKCC1 or KCC2. The intensity of interaction between SPAK and NKCC1 or KCC2 increased markedly after oxygen deprivation, and SPAK overexpression strengthened these interactions. The [Cl^-^]_i_ of hippocampal neurons exhibited corresponding and interesting fluctuations under different conditions. On the basis of these results, together with those from previous studies, we speculate that SPAK is involved in pathophysiologic changes in acquired epilepsy through adjustment of [Cl^-^]_i_ in mouse hippocampus neurons.

MTLE, the most common form of epilepsy in adults, is generally intractable and is suspected to result from recurrent excitation [29]. We used a PISE mouse model to mimic the effects of MTLE on GABA and the corresponding adaptor protein. The PISE mouse model is well-suited for such study for several reasons. First, the natural disease progression, symptoms, and even poor sensitivity to certain drugs in the model are in line with MTLE and are clinically relevant [29,30,31]. Second, this animal model of the disease shares similar neuropathologic features of MTLE in humans. In adults, SE induces a complete re-organization of neural networks, involving cell death, axonal growth, and glutamatergic neosynapse formation, leading to an increased glutamatergic drive [2]. This, in turn, reduces the threshold of seizure generation and thus contributes to the onset of seizures. Furthermore, the PISE model used in this study also exhibited inverse function of GABA signaling [32]. There is evidence that seizures beget seizures and that GABA signaling plays a central role in this phenomenon [2]. Accumulated evidence [10,11,12], including our previously reported findings [9], suggests that a variety of brain insults, including PISE, induces change in the expression of cation-chloride cotransporters KCC2 and NKCC1, resulting in intracellular chloride accumulation and reappearance of immature, depolarizing synaptic responses to GABA(A) receptor activation. This progression may contribute considerably to the neuronal hyperexcitability underlying epileptogenesis. Therefore, alterations in the expression of NKCC1 and KCC2 likely play a crucial role in the process of GABA signaling transformation that follows brain insult.

SPAK, acting as an indirect regulator of [Cl^-^]_i_, is involved in the activation of NKCC1 and inhibition of KCC2 in kidney, intestine, and other organs [13,14,15,16,17,18]. We hypothesized that SPAK plays similar roles in the brain and is related to epileptogenesis. There has been no such report to date. Also, there is no clear evidence showing that SPAK is specifically expressed in cortical or hippocampal neurons, nor are there reported data supporting correlation between SPAK and epilepsy, though it has been reported [33,34,35] that SPAK is abundantly expressed in brain tissue. We therefore verified the 2 prerequisites for this study: SPAK expression in mouse hippocampus neurons and SPAK co-expression with NKCC1 and KCC2. We then determined a long-term expression profile of SPAK in the mouse hippocampus.

The results of this study suggest that the expression of SPAK is altered in the brains of mice with pilocarpine-induced epilepsy. The hippocampal CA1 and CA3 regions, especially the former, are vulnerable in the PISE model [36]. In our study, immunohistochemical analysis showed the post-epilepsy expression levels of SPAK in the CA1 and CA3 regions to be significantly increased post-epilepsy induction. This elevation was moderate on day 1 after SE and higher on day 14, with expression trending downward by day 45, although the expression level was still higher than in control groups in the CA1 region. The statistical difference between the CA1 and CA3 regions can be explained by the increased sensitivity and vulnerability of the CA1 region to the insult. Because the CA1 and CA3 regions of the mouse hippocampus were too small to segregate, we analyzed the entire hippocampus when performing Western Blotting and real-time PCR to avoid tissue isolation bias and fluctuations. Overall, the results obtained are as expected. Interestingly however, the expression pattern observed for SPAK in the PISE mouse model is consistent with that of NKCC1 and contrasts with the profile of KCC2 reported previously findings [9]. Further *in vitro* study will allows us to investigate any implications and the mechanisms underlying the co-alteration of SPAK, NKCC1, and KCC2.

SE and stroke are primary examples of common brain injuries that can lead to the development of acquired epilepsy [37]. Studys have confirmed [38,39,40,41] that ischemia including oxygen-glucose deprivation induced KCC2 down-regulation in the hippocampal (*in vitro* or *in vivo*) accompanied by long-lasting [Cl^-^]_i_ elevation or a sustained depolarizing E_Cl_ shift, which might contribute to hyperexcitability and epileptiform discharges. As described previously, ischemia shares some pathophysiologic and molecular mechanisms with SE during epileptogenesis [37], and may involve the reversal of GABA system function. However, the more important pathologic process of SE is hypoxia due to suffocation rather than ischemia. Therefore, we chose oxygen deprivation to simulate the pathophysiologic process of SE *in vitro*. The results confirmed up-regulation of SPAK and NKCC1 and down-regulation of KCC2 in cultured hippocampal neurons after oxygen deprivation, which are consistent with the results of the *in vivo* PISE model. In addition, we found [Cl^-^]_i_ increased in hippocampal neurons following oxygen deprivation. It appears that oxygen deprivation facilitates functional reshaping of the GABA system, and selection of the *in vitro* model is supported.

Although the expression level of SPAK co-altered with NKCC1 and KCC2, we did not observe changes in the expression level of NKCC1 or KCC2 following SPAK over expression in primary cultured hippocampal neurons under hypoxic or normal conditions. Therefore, NKCC1 and KCC2 expression levels are independent of SPAK, as previously reported [42]. However, it should be noted that oxygen deprivation may also affect the Na/K pump by phosphorylation and therefore results in higher [Cl^-^]_i_ elevation. According to the present study, [Cl^-^]_i_ in cultured hippocampal neurons was reduced following SPAK overexpression, and hypoxia conditioning combined with SPAK over expression elevated it significantly than it in neurons only treated with oxygen-deprivation. These results support reasoning that SPAK must play an important role in regulating [Cl^-^]_i_ in cultured hippocampal neurons. SPAK is bound to have a certain relation with CCCs in the neurons.

Our study demonstrated that SPAK interacts with NKCC1 in cultured hippocampal neurons. Previous experiments performed both *in vitro* and *in vivo* have shown that binding of SPAK is a prerequisite to phosphorylation and activation of this co-transporter [43]. The membrane-associated protein CCCs are phosphorylated by activated SPAK, after which activation of ion transporters changes [34]. CCCs contain an RFx[V/I] motif. At the core of this region, AATYK fits into a pocket of the PF2 region of SPAK, may be the interactor molecule for SPAK [18]. We surmise that similar physiological and biochemical responses occurred in cultured hippocampal neurons. There is considerable evidence for interaction of SPAK with NKCC1. In *Xenopus laevis* oocytes, the phosphorylation of Thr^211^ and Thr^206^ (mouse sequence) is essential for NKCC1 activation, and SPAK/OSR1 must bind to one of these sites prior to activating NKCC1 [44,45]. Addition or removal of a single residue abrogates SPAK activation of NKCC1 [43]. Otherwise, NKCC1 activity is inhibited directly or indirectly by NKCC1 or SPAK dephosphorylation, respectively [46].

Peripheral nerve injury results in increased NKCC1 activity, not as a result of increased co-transporter expression but rather as a result of increased phosphorylation of the co-transporter [47]. Another study showed a reduction in NKCC1 activity in dorsal root ganglion neurons isolated from SPAK knockout mice [48]. On these grounds, we infer that NKCC1 is activated through binding with SPAK in hippocampal neurons, especially following hypoxia conditioning. Although KCC2 was detected solely via co-IP in oxygen-deprived pGC-FU-Stk39-GFP neurons, interaction between SPAK and KCC2 is not ruled out. Yeast two-hybrid analysis revealed that the N-terminus of KCC2 interacts with SPAK [34]. By use of heterologous expression of KCC2 in *Xenopus laevis* oocytes, a dominant-negative effect of SPAK on KCC2 function was detected [34], as expression of kinase-dead SPAK significantly increased KCC2 activity upon hypotonic stimulation [49]. Moreover, tyrosine phosphorylation of KCC2 is likely to play a key role in regulating the degradation of KCC2, a process that may be responsible for the pathologic loss of KCC2 function that is evident in SE and other forms of epilepsy [50,51]. This might explain why KCC2 expression decreased and was difficult to detect via co-IP in our present and previous research [9] studies. It may also account, in part, for the observed [Cl^-^]_i_ increase under conditions such as oxygen-deprivation.

Regarding the exact relationship between CCCs and [Cl^-^]_i_ in neurons, aside from a few reports [52,53], most reports have indicated that KCC2 is activated and extrudes chloride following dephosphorylation and that is loses activity following phosphorylation, whereas NKCC1 exhibits properties and function opposite to those of KCC2. In summary, beside altering NKCC1 and KCC2 expression levels, oxygen deprivation results in up-regulation of endogenous SPAK expression and activity in cultured hippocampal neurons. Active SPAK in turn binds to and phosphorylates CCCs. Up-regulated phosphorylated NKCC1 mediates chloride influx, while KCC2 is down-regulated, inhibited, and extrudes less chloride. Thus [Cl^-^]_i_ excess is an additive effect of CCCs and SPAK in neurons following oxygen deprivation. However, SPAK overexpression expands oxygen deprivation effects in cultured hippocampal neurons, as elevated active SPAK participates in the regulation process, shown also in the results of this study. The [Cl^-^]_i_ decline in neurons overexpressing SPAK under normoxic conditions could be associated with activated KCC2 and inhibited NKCC1 resulting from increased inactivity of exogenous SPAK. In the process, overexpressed SPAK remains dephosphorylated and without activity. It activates KCC2 and inhibits NKCC1, therefore the lowest [Cl^-^]_i_ and strongest MQAE fluorescence were observed in the study.

In conclusion, our present study showed that SPAK is up-regulated during various periods following PISE in mice. Future studies on the relationships between SPAK, CCCs, the regulator [Cl^-^]_i_ in neurons, and [Cl^-^]_i_ itself *in vitro* are needed to clarify the role of SPAK in epilepsy. SPAK is recognized as a regulating kinase for the [Cl^-^]_i_ in hippocampal neurons and may be involved in the plasticity of GABA signaling function in acquired epilepsy. Thus, it seems quite reasonable to postulate that SPAK may become a new therapeutic target for acquired epilepsy, especially GABA-related drug resistant epilepsy. However, there are limitations that remain to be consummated from this study. First, knock-down of SPAK with siRNA or substitution of key phosphorylation sites would further support our findings and implications for targeting SPAK as an epilepsy therapy. Second, it should be noted that oxygen deprivation also affects the Na/K pump, which may in turn affect the driving force for the cotransporters. Another limitation is that we did not measure Na/K pump activity. Therefore, future studies should be conducted to investigate whether oxygen-deprivation affects the co-transporters through the Na/K pump.
